# *Coxiella burnetii* Shedding in Milk and Molecular Typing of Strains Infecting Dairy Cows in Greece

**DOI:** 10.3390/pathogens10030287

**Published:** 2021-03-03

**Authors:** Emmanouil Kalaitzakis, Tiziano Fancello, Xavier Simons, Ilias Chaligiannis, Sara Tomaiuolo, Marianna Andreopoulou, Debora Petrone, Aikaterini Papapostolou, Nektarios D. Giadinis, Nikolaos Panousis, Marcella Mori

**Affiliations:** 1 Clinic of Farm Animals, Faculty of Veterinary Medicine, School of Health Sciences, Aristotle University of Thessaloniki, 54627 Thessaloniki, Greece; ekalaitzakis@vet.auth.gr (E.K.); ngiadini@vet.auth.gr (N.D.G.); panousis@vet.auth.gr (N.P.); 2 Sciensano, Veterinary Bacteriology, Scientific Directorate Infectious Diseases in Animals, 1180 Brussels, Belgium; Tiziano.Fancello@sciensano.be (T.F.); Sara.Tomaiuolo@sciensano.be (S.T.); Deborah.Petrone@sciensano.be (D.P.); 3 National Reference Centre *Coxiella burnetii*, Sciensano, 1180 Brussels, Belgium; 4 Sciensano, Veterinary Epidemiology, Scientific Directorate Epidemiology and Public Health, 1180 Brussels, Belgium; Xavier.Simons@sciensano.be; 5 Centre for Infectious Diseases, Institute of Parasitology, University Leipzig, An den Tierkliniken 35, 04103 Leipzig, Germany; marianna.andreopoulou@vetmed.uni-leipzig.de; 6 Laboratory of Microbiology and Infectious Diseases, Faculty of Veterinary Medicine, School of Health Sciences, Aristotle University of Thessaloniki, 54124 Thessaloniki, Greece; vetkatiapap@gmail.com

**Keywords:** *Coxiella burnetii*, Q fever, genotyping, epidemiology, BTM, Greece

## Abstract

Ruminants are considered the commonest animal reservoir for human infection of *Coxiella burnetii*, the Q fever causative agent. Considering the recently described importance of human Q fever in Greece, we aimed at providing the first comprehensive direct evidence of *C. burnetii* in dairy cows in Greece, including the genetic characterization of strains. The 462 examined dairy farms represented all geographical areas of Greece. One bulk tank milk sample was collected from every farm and tested for the presence of *C. burnetii*. Molecular genotyping of strains, performed directly on samples, revealed the existence of two separate clades characterized by single nucleotide polymorphism (SNP) genotypes of type 1 and type 2. The two clades were clearly distinguished in multiple locus variable-number tandem repeat analysis (MLVA) by two discriminative loci: MS30 and MS28. Whereas MLVA profiles of SNP-type 2 clade were closely related to strains described in other European cattle populations, the MLVA profile observed within the SNP type 1 clade highlighted a peculiar genetic signature for Greece, related to genotypes found in sheep and goats in Europe. The shedding of *C. burnetii* bearing this genotype might have yet undefined human epidemiological consequences. Surveillance of the genetic distribution of *C. burnetii* from different sources is needed to fully understand the epidemiology of Q fever in Greece.

## 1. Introduction

*Coxiella burnetii*, the causative agent of Q fever in humans, is a well-documented intracellular gram-negative γ-proteobacterium, prevalent in the Mediterranean area [1], but also recognized as endemic worldwide, except in New Zealand [2]. The bacterium is pleomorphic and exists in two forms along its developmental cycle: the large (LCV) (>0.5 µm) and the small cell variants (SCV) (0.2–0.5 µm). The SCV is the form with enhanced stability in the environment and the form ensuring transmission through the aerosol route [3].

*C. burnetii* is a pathogen detected in various species throughout the animal kingdom [4], but ruminants (sheep, goats, and cattle) are considered the most common animal reservoir for human infection [5]. A variety of other mammals, birds, and arthropods can be infected, thus contributing to the maintenance of the bacterium in the environment [6]. In ruminants, the infection may result in abortions during which large amounts of bacteria are shed in placenta and birth fluid. Contaminated particles in the form of aerosols are considered the main route of transmission to humans [7]. In humans, Q fever can manifest as an acute or chronic illness. Acute disease is typically a self-limiting, febrile illness during which pneumonia or hepatitis can occur, whereas chronic disease, although rare, is a severe illness that usually manifests as endocarditis and occasionally as vascular infection, osteomyelitis, or chronic hepatitis [7]. In cattle, Q fever is associated with late abortions and reproductive disorders such as premature birth, delivery of dead or weak offspring, metritis, and infertility [8], although in the majority of cases, infection remains subclinical and asymptomatic [9]. *C. burnetii* is therefore a cause of economic losses in cattle and is a public health concern in general.

In recent years, interest has increased in *C. burnetii* mainly due to the number and proportion of recent outbreaks in Europe concerning human infections [10,11].

Epidemics of Q fever are partly an example of the interactions between disease burden and agricultural practices and offer information on risks and drivers of a zoonotic disease at the livestock–human interface [12]. Investigations in the cattle population and the knowledge of the infection status in herds are important to understand the epidemiology of the pathogen in a specific area/country. In dairy cattle, milk, which is one shedding route of *C. burnetii*, is easy to collect and animals with bacterial loads in milk can be easily identified in dairy herds [13]. Moreover, infected dairy cattle without clinical signs can shed *C. burnetii* in milk for several months [14]. Owing to the property of simplicity in sampling combined with the relevance for Q fever diagnosis, bulk tank milk (BTM) samples are appropriate for monitoring *C. burnetii* infection at the herd level [9].

Fast fingerprinting of *C. burnetii* isolates using molecular genotyping tools is essential for epidemiological surveys from different geographical areas or hosts. Harmonized schemes for typing are yet to be agreed upon for *C. burnetii,* but multiple locus variable-number tandem repeat analysis (MLVA) is depicted as being highly discriminatory and single nucleotide polymorphism (SNP) typing as the best option in case of fairly loaded samples [15,16]

Greece poses an interesting challenge for *C. burnetii* investigation since it has a very large population of dairy small ruminants: a total of 12,626,520 animals allocated in 154,926 farms, under very diverse farm conditions [17]; concerning dairy cow herds, there are 2637, with around 171,000 dairy cows. Regarding dairy cattle, there are farms in different geographical areas, including continental Greece and islands with hot climate conditions. Moreover, clinical human Q fever cases are being steadily recorded, with almost 200–250 new registered cases every year [18]. Another challenge is that Greece neighbors non-EU countries with large ruminant populations, in which initiatives on animal disease control are more difficult to apply and harmonize with EU legislation. Finally, the consequences of global climate change favoring arthropods’ activity for a longer time yearlong poses a special interest on *C. burnetii*. Despite the aforementioned importance of *C. burnetii*, there is a lack of studies concerning infection with this pathogen in animals in Greece. There are a couple of serological studies in sheep and goats [19,20] and one serological study in dairy cows [21]. Molecular characterization of strains was approached in a single recent work, which examined aborted sheep fetuses [22]. There is no information available on the genotypic diversity of the strains circulating all over the country, data important for both surveillance and epidemiological investigation.

The purpose of this study was to assess *C. burnetii* prevalence and infection in dairy cattle herds in all geographical areas of Greece and to apply MLVA and SNP schemes to characterize the genetic diversity of the *C. burnetii* strains circulating in the country as a first step to establish a link with potential sources of human infection.

## 2. Material and Methods

### 2.1. Sample Collection

A total of 462 bulk tank milk (BTM) samples were collected from 462 dairy cattle herds (Holstein, unique breed of all dairy cows in Greece). Sampling was performed from December 2017 to October 2018. Sampled herds were selected to be as representative as possible of the dairy cattle herds in Greece. Sampling was performed with stratified random selection based on geographical position and the size of the herds. Special effort was made to proportionally sample all geographical areas of the country having dairy cattle herds, including continental Greece and islands. From each area, all types of herds were sampled, regardless of herd size and management practices. In special concern to cattle population, more herds were sampled from the areas with a bigger population from each farm, 50 mL of milk was collected directly from the bulk tank into a sterile plastic tube, transferred under refrigeration (electric powered portable refrigerator) to the Farm Animal Clinic of the Aristotle University of Thessaloniki, and stored at −20 °C till they were sent, under dry ice condition, to Sciensano, Brussels, for analysis. All samples were kept refrigerated in −20 °C till thawed for analysis.

### 2.2. DNA Extraction and Diagnostic Real Time-PCR

DNA was extracted directly from 200 µL BTM using the MagMax™ Isolation Kit (Thermofisher, Waltham, MA, USA) according to the manufacturer’s instructions. We tested 1/50 of the eluted DNA for the presence of *C. burnetii* DNA with a PCR (rt-PCR) reaction targeting the *IS1111* repetitive element [23]. The PCR protocol, primers, and cycling conditions were as those previously described [24]. The rt-PCR assay was performed using a 7500 Real-Time PCR System (Thermofisher). The results from positive samples, showing a typical amplification curve, are expressed as cycle threshold (Ct) values. Ct values below 40 were considered to be positive [24].

### 2.3. MLVA and SNP Typing

DNAs were challenged to MLVA on 13 markers (MS03, MS12, MS21, MS22, MS30, MS36, MS27, MS28, MS31, MS23, MS24, MS33, and MS34). Sequences of primers were derived from previous works and their improved versions [15,25,26]. Amplified fragments were analyzed by capillary electrophoresis on a CEQ 8000 Genetic Analysis System (Beckman Coulter, Indianapolis, IN, USA) and their exact length was measured at the nucleotide level by the concomitant run of an internal base-ladder. Only MS21 was run on 1% agarose gel electrophoresis and quantified according to a DNA ladder. Successful amplifications for MLVA were variable but mostly obtained when rt-PCR provided a result with a Ct value <30. Single-nucleotide genotyping was performed exactly as described previously [16]. The data were submitted to http://microbesgenotyping.i2bc.paris-saclay.fr/ (latest accessed on 2 March 2021) public repository.

### 2.4. Statistical Analyses and Cartography

Sample size estimation was based on Epitools (https://epitools.ausvet.com.au/, accessed on 2 March 2021). For the overall apparent shedding herd prevalence, the estimate was calculated considering an estimated true proportion of 0.3, precision of 0.05, and 95% confidence interval (CI). Prefecture prevalence was defined only for a sample size representing the last 15% of herds. Upper and lower CI limits were calculated with the Wilson method for a confidence level of 95%.

Clustering analyses were performed using BioNumerics version 6.6 software (Applied Maths, Sint-Martens-Latem, Belgium). Minimum spanning trees were built on the categorical data of the Greek dataset (this work) or available public data (http://microbesgenotyping.i2bc.paris-saclay.fr/, accessed on 2 March 2021). The correlation of SNP data with each MLVA marker value was calculated using Spearman’s rank correlation coefficient with a significant value set at *p* < 0.05. Maps were created using R software (version 3.6.1, the R Foundation for Statistical Computing, Vienna, Austria) and the tmap package [27].

## 3. Results

### 3.1. Shedding Herd Prevalence and Bacterial Load in Bovine BTM Milk

In total, 156 of the 462 sampled farms were positive for *C. burnetii* DNA, showing an overall apparent shedding herd prevalence of 33.8% (CI 29.6–38.2) throughout the country. All but the Ionian Islands region had at least one positive farm, with varying degrees of prevalence (Table 1).

**Figure 1:**
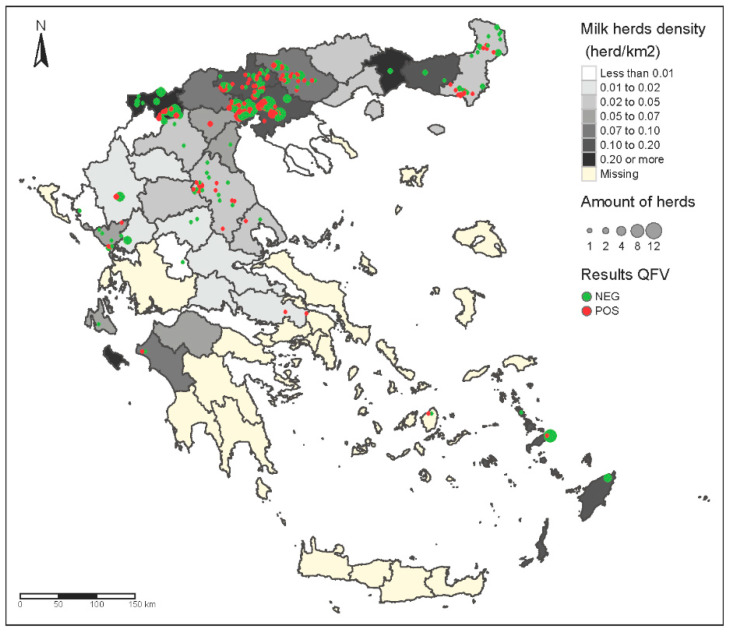
Geographical distribution of positive and negative *C. burnetii* samples and depiction of milk herd density in Greece.

The geographical distribution of positive samples revealed that in almost all sampled places, we found *C. burnetii* shedding and positive farms (Table 1, Figure 1). Prevalence was higher in Thrace, where the density of dairy farms is not particularly high (0.02–0.05 herds/km^2^) in comparison to other areas, like Central Macedonia (Figure 1).

### 3.2. SNP and MLVA Genotyping

**Figure 2:**
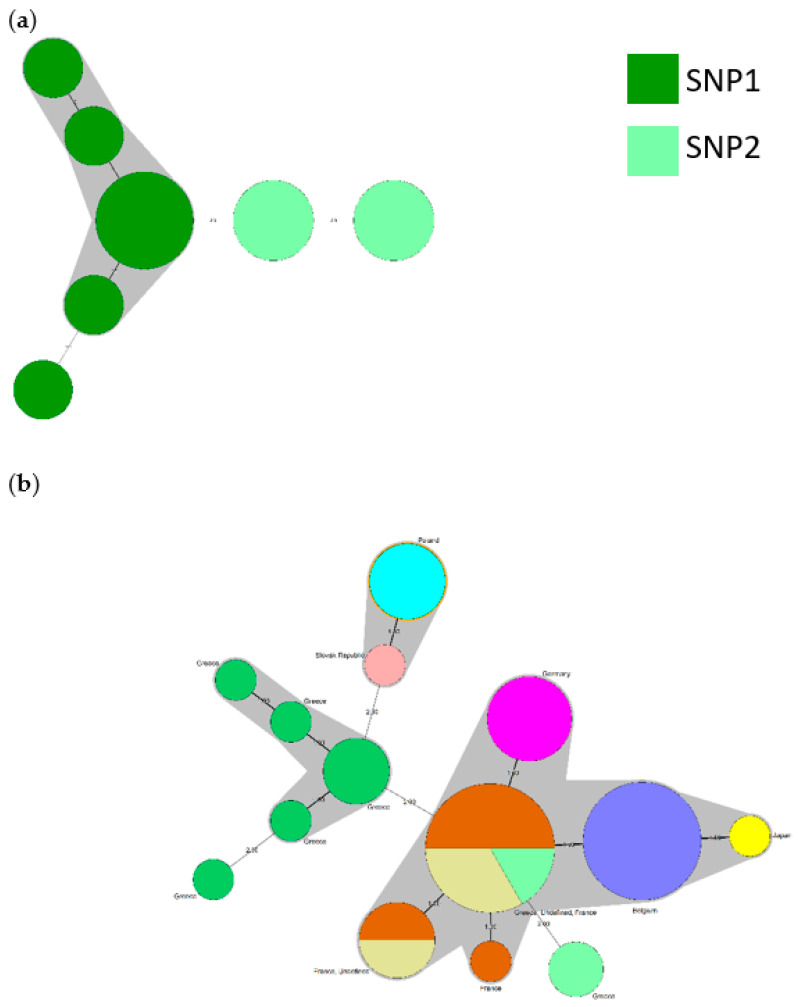
(**a**) Minimum spanning tree representation of combined multiple locus variable-number tandem repeat analysis and single nucleotide polymorphism (MLVA-SNP) profiles of Greek cows’ milk samples. Six-locus Greek MLVA profiles, with an acceptable missing value of two, are represented. Clustering was performed with Bionumerics. Circles outline the genetic MLVA profiles of strains and color the different SNP profiles: SNP type 1 (yellow) or SNP type 2 (blue). Numbers on the connecting lines refer to the number of MLVA markers differing between samples. The grey area agglomerates strains with a single MLVA locus difference. The size of the circles is proportional to the number of strains bearing the same genetic profile. (**b**) Minimum spanning tree representation of MLVA combined with SNP profile of Greek cows’ milk samples, together with cattle profile described for Europe (retrieved from http://microbesgenotyping.i2bc.paris-saclay.fr/, accessed on 2 March 2021).

**Figure 3:**
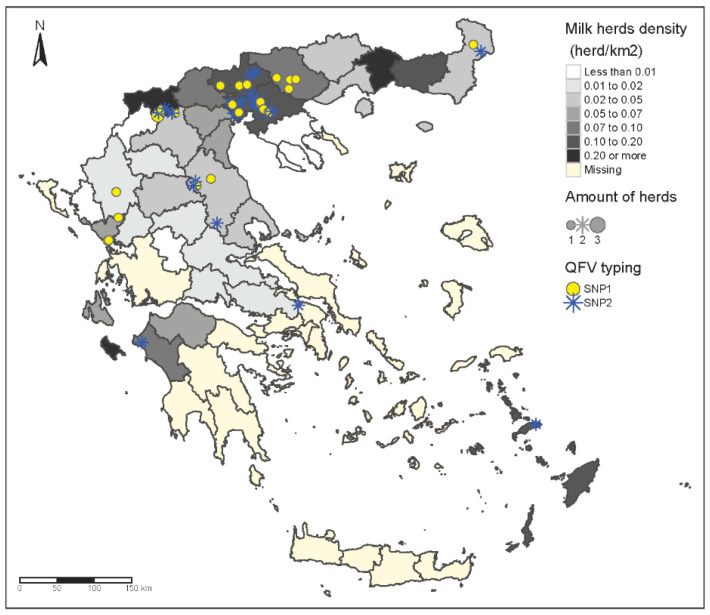
Geographical distribution of two different SNP genotypes isolated and depiction of milk herd density in Greece.

Genotyping of *C. burnetii* was performed by SNP analysis in 50/59 positive samples that had favorable Ct and were randomly selected across the country. The analysis revealed the existence of two separate SNP genotypes: genotype type 1 and type 2 (Figure 2a). Both genotypes were isolated in geographical areas with high dairy farm density and in areas with many positive samples, like Central Macedonia (Figure 3). Interestingly, SNP genotype 2 was the only type isolated in south continental Greece (Peloponessos, Viotia), and in Cyclades and Dodecanese islands, which are areas where the number of dairy farms is low. The SNP genotype 1 was the only isolated genotype in Western Greece, in the region of Epirus (Figure 3). MLVA13 analysis was conducted on the highly *C. burnetii* DNA-charged samples (22), obtaining partial MLVA profiles for 21 and a complete MLVA for 1 sample (see Supplementary Materials).

Clustering of MLVA6 genotypic data illustrated with the minimum spanning tree again highlighted the presence of two separate genetic groups corresponding to the two observed SNP types (Figure 2a). A perfect correlation between SNP data and the number of repeats in the various MLVA markers was observed for MS30, which was always associated with five repeats for SNP type 1 and six for SNP type 2 (*p* = 0.002), and MS28, which was always associated with three repeats for SNP type 1 and seven for SNP type 2 (*p* < 0.001). Clustering of MLVA6 data with cattle MLVA6 data retrieved from the public repository showed that Greek cattle samples belonging to SNP type 2 are closely related to strains characterized in Belgium [24], France, Germany, and Japan, differing from them in none or a single locus. Conversely, Greek cattle samples having an SNP type 1 profile cluster significantly apart from the Belgium/France/Germany/Japan cluster and the Poland/Slovak Republic cluster (Figure 2b).

**Figure 4:**
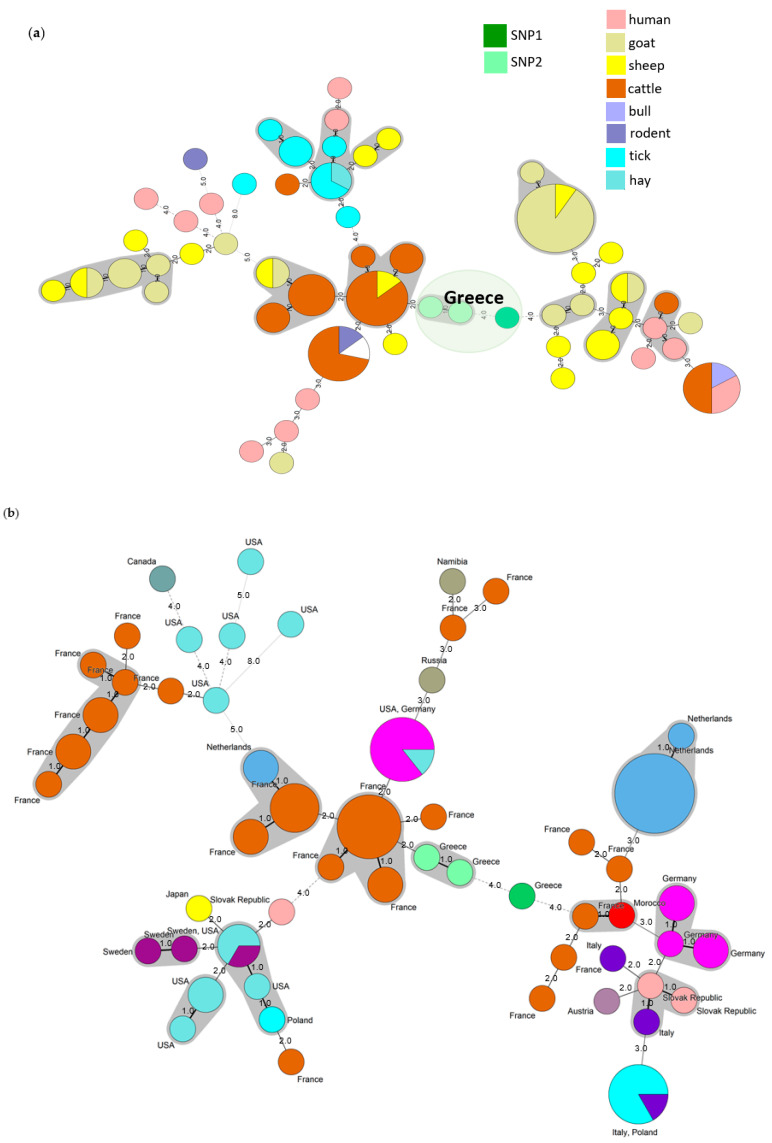
Minimum spanning tree representation of combined MLVA-SNP profiles of three Greek cow milk samples (B78, TM16, and FA59) together with European strains deposited in the public database (http://microbesgenotyping.i2bc.paris-saclay.fr/, accessed on 2 March 2021). There are 13-locus Greek and EU MLVA profiles, with an acceptable missing value of two, represented. Representation of the clustering according to (**a**) host species and (**b**) geographical location. Circles outline the genetic MLVA profiles of strains. Numbers on the connecting lines refer to the number of MLVA markers differing between samples. The grey area agglomerates strains with a single MLVA locus difference. The size of the circles is proportional to the number of strains bearing the same genetic profile. Different colors represent the different countries and are indicated close.

A deeper analysis using three Greek strains (B78, TM16, and FA59) characterized by a larger number of markers (MLVA 12) and strains of various host origins present in the public repository illustrated that Greek strains have a peculiar genetic profile. While B78 and TM16 (SNP type 2) are similar to the European cattle strains (with only two- to three-marker difference), the FA59 sample has a Greece-specific MLVA profile with genetic characteristics in between strains in European cattle and European goat/sheep (each four-marker difference from the nearest bovine or goat/sheep strain) (Figure 4a,b). This completely new genotype might probably characterize also all the other SNP type 1 Greek strains found in this work and for which a full MLVA could not be established for technical reasons. The new genotype is geographically spread in Northern Greece and other areas with intensive farming (Figure 3).

## 4. Discussion

This study is the first to use molecular techniques to examine milk samples to report the prevalence of *C. burnetii* in dairy cows in Greece. It is also the first to examine samples collected nationwide. Almost 18% of Greek dairy herds were investigated, which is a high number and representative of the dairy cattle industry in the country. Special effort was made to cover all geographical regions having dairy cattle and to proportionally sample them; most samples were obtained from Central Macedonia, the area with a larger number of dairy farms. Notably, an adequate number of farms from islands and remote places with a very low dairy cow population was also sampled. Thus, the results depict *C. burnetii* shedding in dairy cow farms of the whole country.

The overall prevalence of 33.7% revealed that *C. burnetii* is common in the dairy cattle population in Greece. This finding is consistent with the reported prevalence in other countries, confirming that *C. burnetii* shedding through milk is widespread in dairy cattle herds in different countries. Reports from other countries like the USA, the Netherlands, Spain, Hungary, Portugal, Iran, and Poland revealed a wide range of prevalence, reporting shedding of *C. burnetii* through cattle milk of between 18.8% and 94.3% [9,28,29,30,31,32,33]. The reported *C. burnetii* prevalence of 33.7% in Greek dairy cattle is around the mid-range of the other countries, but still can be considered as high, since almost one out of three farms host active *C. burnetii* shedders. Positive farms were recorded in all geographical areas tested, which covered all the regions of the country, showing that active *C. burnetii* shedders are spread in the whole country. There are no previous national or regional data with which to compare, but the results are consistent with those of other European countries [29,30,33,34]. The high *C. burnetii* DNA prevalence in dairy cattle could be explained by the long-time excretion of *C. burnetii* through milk, which, in cattle, can extend for several months compared with the shorter excretion periods reported for sheep and goats [35].

In cattle, *C. burnetii* is shed in milk and other secretory routes [35], so examining milk is adequate for herd and regional-wide monitoring. Single BTM samples were selected from each farm, as they are easy to collect, helpful for scanning a large number of farms, and can provide valuable epidemiological data. Concerning the performed molecular methods, SNP genotyping of *C. burnetii* provides informative epidemiological insight and is particularly suitable for direct typing of strains from veterinary materials with very limited bacterial load [16]. Since the first proposal of a panel to be used for the genotyping of *C. burnetii* [15], several studies have been conducted throughout Europe to increase knowledge of the circulating strains. MLVA, in particular, presents the highest discriminatory power compared to multi-sequence space typing (MST) [36,37]. Furthermore, typing by MLVA could be standardized between laboratories, although a harmonized scheme and a genetic strain nomenclature for *C. burnetii* is yet distinct between groups.

In Greece, data were lacking concerning isolates from cows, other ruminants, and even humans; therefore, the genotypes of *C. burnetii* revealed here cannot be compared with previous ones. Recently, information regarding *C. burnetii* diversity in Greece was restricted to the description of a single strain from a human sample, a strain closely related to MST 18 [38], and ruminants strains isolated from the abortion tissues of sheep and goats [22]. Notably, the sampled dairy cattle in the present work were from the regions of the isolated *Coxiella* strains of sheep abortions. Comparison of our data with MLVA data from this previous work [22] is difficult due to the different methods used to calculate the repeats and the different panel of loci used in the two studies. As described, the MLVA profiles identified from sheep abortion material are different from the genotypes isolated in the present work. The lack of harmonization of the MLVA panels and the amplified loci lead to results that are difficult to compare among laboratories [25]. The current study is the first attempt to genotype *C. burnetii* strains in dairy cattle in Greece; it should be continued by adding more samples and data, especially from small ruminants all over Greece. Moreover, it would be advisable to compare strains from animals with clinical symptoms (like abortion, metritis, and low fertility) against strains of healthy animals.

Besides genotypes already described in Europe (the SNP-type 2 strains), a novel profile was identified in the SNP type 1 genotype group. In particular, the fully characterized strain FA59 consisted of a peculiar genetic profile. This strain has a Greece-specific MLVA profile with genetic characteristics between European cattle and European goat/sheep strains. This finding is interesting concerning the closer relation of sheep and goats’ strains to human infection [39], and further investigation would expand our understanding of the epidemiology of these strains in the national population. This new strain might have more virulent characteristics and, even though found in cattle, might be important for human outbreaks, as are small ruminant strains. There are some unpublished data regarding a new strain isolated from humans with chronic Q fever clinical disease in Greece that is under investigation (personal communication, National Health Surveillance Institute).

As reported earlier [40], genetic diversity among *Coxiella* strains infecting the European dairy cattle population is low, so the existence of a new genotype proved by the present study is interesting. Novel genotypes were described earlier [34,39,41,42,43], but in these studies, they were isolated from one sample each [43] or were sporadically and locally found and represented a low percentage of isolates [39]. In contrast, in our present work, the new genotype SNP-type 1 clade was isolated in almost half of the positive samples and was widespread in the country, especially in the areas with high dairy cattle farm density (Macedonia and Thrace), while it was the only genotype isolated from the western part of the country (Epirus). More MLVA data are necessary to obtain a more comprehensive image of the complete population structure of *C. burnetii* in Greece.

Clinical manifestation of *Coxiella* is under investigation because different strains and genotypes cannot be easily related to reproductive problems, since very few virulence-associated genes are annotated and virulence mechanisms of *C. burnetii* are still poorly understood [44]. Available data are not consistent since there is proven evidence of reproductive disorders attributed to *Coxiella* shedding in dairy cows [13,31,45] and, in contrast, absence of reproductive problems in herds with established *C. burnetii* infection [9,29,46]. The absence of a permanent relation between reproductive problems and bacterial DNA may indicate that a herd may carry *C. burnetii* for a long period without developing any major clinical signs [14], or that there are strains and genotypes with varying pathogenicity and special research attention is needed for them. Moreover, aerosol transmission, environmental stability, and a very low infectious dose [47] make *C. burnetii* a challenging pathogen for diagnosis and elimination. Concerning the results of the present study, the new genotype being isolated, which seems closer to sheep and goat’s genotype, is of special interest, since the *Coxiella* infections cause more frequent and more serious symptoms and diseases (abortion and reproductive disorders) in small ruminants than in cattle, and are closely related to human infection and have been implicated in human outbreaks [10]. The investigation of the reproductive and disease history of these positive herds would possibly elucidate the relation of this *Coxiella* genotype with clinical reproductive disorders and help evaluate the possible clinical relevance of the new peculiar isolated genotype.

When cattle are on the same farm with other species, especially sheep and goats, there is a higher prevalence and within-herd seroprevalence of *C. burnetii* infection [14,48]. The large population of small dairy ruminants, the vicinity with dairy cattle herds, the newly identified cattle strain, and the constant number of cases in humans indicate the necessity of investigating the prevalence of *C. burnetii* with advanced molecular techniques in small ruminants. Surveillance of the genetic distribution of *C. burnetii* from different sources is needed to fully understand the epidemiology of Q fever in Greece.

## 5. Conclusions

This paper reports the first comprehensive *C. burnetii* prevalence investigation in dairy cows in Greece with advanced molecular techniques. The results revealed that *C. burnetii* is widespread in dairy cattle herds in Greece, showing a similar situation to that described in other countries. Low genotyping diversity was recorded in the dairy cows population, but a new genotype, SNP type 1, with a peculiar MLVA profile was isolated that is more genetically related to isolated genotypes of sheep and goats in Europe. This situation indicates the need for further studies on the epidemiological consequences of *C. burnetii* shedding in the milk of cattle and especially further indicates the importance of molecular investigation regarding sheep and goats in the country since, until now, there were no relevant data available. The collection of such data and their comparison with data deposited in international databases will help toward both continuing active surveillance and strain genotyping of the pathogen, as well as to better understanding the epidemiology of the disease across Europe.

## Figures and Tables

**Table 1:** Greek cattle herds, geographical distribution, tested samples and apparent prevalence.

Province	Prefecture	Total Number of Herds	Number of Tested Herds	Number of Tested Samples	Number of Positive Samples	Apparent Shedding Herd Prevalence	CI (Lower-Upper 95%)
Thrace	Evros	123	35	35	15	0.429	0.280–0.591
Epirus	Arta	30	5	5	0	0.000	0.000–0.435
	Thesprotia	5	1	1	0	0.000	0.000–0.794
	Ioannina	54	7	7	2		
	Preveza	53	10	10	3	0.429	0.280–0.591
Thessaly	Karditsa	50	2	2	0		
	Larissa	217	23	23	12		
	Magnisia	53	3	3	1		
Central Macedonia	Imathia	51	2	2	2		
	Thessaloniki	470	174	174	64	0,368	0.300–0.442
	Kilkis	287	60	60	25	0.417	0.300–0.543
	Serres	308	46	46	16	0.348	0.227–0.492
Western Macedonia	Florina + Kozani	351	68	68	10	0.147	0.082–0.250
Central Greece	Evritania	10	1	1	0		
	Viotia	57	2	2	2		
Peloponesse	Ilia	170	2	2	1		
Cyclades islands	Naxos	120	5	5	2		
Dodecanese	Rhodes	55	4	4	0		
	Kos	125	10	10	1		
	Leros	26	1	1	0		
Ionian islands	Cephalonia	22	1	1	0		
Total		2637	462	462	156	0.338	0.296–0.382

## Data Availability

The data presented in this study are openly available in http://microbesgenotyping.i2bc.paris-saclay.fr/ (accessed on 2 March 2021), *Coxiella burnetii*, reference number 380 to 401.

## Supplementary Materials

The following are available online at https://www.mdpi.com/2076-0817/10/3/287/s1.

## References

[1] Robbins F.C., Gauld R.L., Warner F.B. (1946). Q fever in the Mediterranean area; report of its occurrence in Allied troops; epidemiology. Am. J. Hyg..

[2] Eldin C., Mélenotte C., Mediannikov O., Ghigo E., Million M., Edouard S., Mege J.-L., Maurin M., Raoult D. (2017). From Q fever to *Coxiella burnetii* infection: A paradigm change. Clin. Microbiol. Rev..

[3] Sandoz K.M., Popham D.L., Beare P.A., Sturdevant D.E., Hansen B., Nair V., Heinzen R.A. (2016). Transcriptional profiling of *Coxiella burnetii* reveals extensive cell wall remodeling in the small cell variant developmental form. PLoS ONE.

[4] Kersh G.J., Lambourn D.M., Raverty S.A., Fitzpatrick K.A., Self J.S., Akmajian A.M., Jeffries S.J., Huggins J., Drew C.P., Zaki S.R. (2012). *Coxiella burnetii* infection of marine mammals in the Pacific Northwest, 1997–2010. J. Wildl. Dis..

[5] Botner A., Broom D., Doherr M.G., Domingo M., Hartung J., Keeling L., Koenen F., More S., Morton D., Oltenacu P. (2010). Scientific opinion on Q fever. EFSA J..

[6] Cutler S.J., Bouzid M., Cutler R.R. (2007). Q fever. J. Infect..

[7] Maurin M., Raoult D. (1999). Q fever. Clin. Microbiol. Rev..

[8] Rodolakis A. (2009). Q Fever in Dairy Animals. Ann. N Y. Acad. Sci..

[9] Muskens J., van Engelen E., van Maanen C., Bartels C., Lam T.J. (2011). Prevalence of *Coxiella burnetii* infection in Dutch dairy herds based on testing bulk tank milk and individual samples by PCR and ELISA. Vet. Rec..

[10] Roest H.I., Ruuls R.C., Tilburg J.J., Naburs Frassens M.H., Klaassen C.H.W., Vellema P., van den Brom R., Dercksen D., Wouda W., Spierenburg M.A.H. (2011). Molecular epidemiology of *Coxiella burnetii* from Ruminants in Q fever outbreak, the Netherlands. Emerg. Infect. Dis..

[11] Georgiev M., Afonso A., Neubauer H., Needham H., Thiery R., Rodolakis A., Roest H., Stark K., Stegeman J., Vellema P. (2013). Q fever in humans and farm animals in four European countries, 1982 to 2010. Eurosurveillance.

[12] Mori M., Roest H.J. (2018). Farming, Q fever and public health: Agricultural practices and beyond. Arch. Public Health.

[13] Guatteo R., Joly A., Beaudeau F. (2012). Shedding and serological patterns of dairy cows following abortions associated with *Coxiella burnetii* DNA detection. Vet. Microbiol..

[14] Piñero A., Ruiz-Fons F., Hurtado A., Barandika J., Atxaerandio R., García-Pérez A. (2014). Changes in the dynamics of *Coxiella burnetii* infection in dairy cattle: An approach to match field data with the epidemiological cycle of C. *burnetii* in endemic herds. J. Dairy Sci..

[15] Arricau-Bouvery N., Hauck Y., Bejaoui A., Frangoulidis D., Bodier C.C., Souriau A., Meyer H., Neubauer H., Rodolakis A., Vergnaud G. (2006). Molecular characterization of *Coxiella burnetii* isolates by infrequent restriction site-PCR and MLVA typing. BMC Microbiol..

[16] Huijsmans C.J., Schellekens J.J., Wever P.C., Toman R., Savelkoul P.H., Janse I., Hermans M.H. (2011). Single-nucleotide-polymorphism genotyping of *Coxiella burnetii* during a Q fever outbreak in The Netherlands. Appl. Environ. Microbiol..

[17] National Statistical Service of Greece (2016). Agriculture and Livestock Census. https://www.statistics.gr/en/home/.

[18] National Public Health Organization (2019). Mandatory Notification System, Reports. eody.gov.gr/en/epidemiological-statistical-data/monthly-data-mandatory-notification-system.

[19] Pape M., Bouzalas E., Koptopoulos G., Mandraveli K., Arvanitidou-Vagiona M., Nikolaidis P., Alexiou-Daniel S. (2009). The serological prevalence of *Coxiella burnetii* antibodies in sheep and goats in northern Greece. Clin. Microbiol. Infect..

[20] Filioussis G., Theodoridis A., Papadopoulos D., Gelasakis A.I., Vouraki S., Bramis G., Arsenos G. (2017). Serological prevalence of *Coxiella burnetiii* n dairy goats and ewes diagnosed with adverse pregnancy outcomes in Greece. Ann. Agric. Environ. Med..

[21] Dovolou E., Tsiligianni T., Vouzaras D., Amiridis G.S. (2011). Prevalence of *Coxiella burnetii* antibodies in bulk milk and blood serum and associations with reproductive indices in cow dairy herds of Central and Northern Greece. J. Hell. Vet. Med. Soc..

[22] Chochlakis D., Santos A.S., Giadinis N.D., Papadopoulos D., Boubaris L., Kalaitzakis E., Psaroulaki A., Kritas S.K., Petridou E.I. (2018). Genotyping of *Coxiella burnetii* in sheep and goat abortion samples. BMC Microbiol..

[23] Klee S.R., Ellerbrok H., Tyczka J., Franz T., Appel B. (2006). Evaluation of a real-time PCR assay to detect *Coxiella burnetii*. Ann. N. Y. Acad. Sci..

[24] Mori M., Boarbi S., Michel P., Bakinahe R., Rits K., Wattiau P., Fretin D. (2013). In vitro and in vivo infectious potential of *Coxiella burnetii*: A study on belgian livestock isolates. PLoS ONE.

[25] Svraka S., Toman R., Skultety L., Slaba K., Homan W.L. (2006). Establishment of a genotyping scheme for *Coxiella burnetii*. FEMS Microbiol. Lett..

[26] Tilburg J.J.H.C., Rossen J.W.A., Van Hannen E.J., Melchers W.J.G., Hermans M.H.A., Van De Bovenkamp J., Roest H.J.I., De Bruin A., Nabuurs-Franssen M.H., Horrevorts A.M. (2011). Genotypic diversity of *Coxiella burnetii* in the 2007–2010 Q Fever outbreak episodes in The Netherlands. J. Clin. Microbiol..

[27] Tennekes M. (2018). tmap: Thematic maps in R. J. Stat. Softw..

[28] Kim S.G., Kim E.H., Lafferty C.J., Dubovi E. (2005). *Coxiella burnetii* in bulk tank milk samples, United States. Emerg. Infect. Dis..

[29] Astobiza I., Ruiz-Fons F., Piñero A., Barandika J., Hurtado A., García-Pérez A. (2012). Estimation of *Coxiella burnetii* prevalence in dairy cattle in intensive systems by serological and molecular analyses of bulk-tank milk samples. J. Dairy Sci..

[30] Gyuranecz M., Dines B., Hornok S., Kovacs P., Horvath G., Jurkovich V., Varga T., Hajtos I., Szabó R., Magyar T. (2012). Prevalence of *Coxiella burnetii* in Hungary: Screening of dairy cows, sheep, commercial milk samples and ticks. Vector Borne Zoonotic Dis..

[31] Anastácio S., Carolino N., Sidi-Boumedine K., Da Silva G.J. (2014). Q fever dairy herd status determination based on serological and molecular analysis of bulk tank milk. Transbound. Emerg. Dis..

[32] Van Engelen E., Schotten N., Schimmer B., Hautvast J., Van Schaik G., van Duijnhoven Y. (2014). Prevalence and risk factors for *Coxiella burnetii* (Q fever) in Dutch dairy cattle herds based on bulk tank milk testing. Prev. Vet. Med..

[33] Nokhodian Z., Feizi A., Moradi A., Yaran M., Hoseini S.G., Ataei B., Hosseini M. (2016). Detection and risk factors of *Coxiella burnetii* infection in dairy cattle based on bulk tank milk samples in center of Iran. Prev. Vet Med..

[34] Santos A.S., Tilburg J.J., Botelho A., Barahona M.J., Núncio M.S., Nabuurs-Franssen M.H., Klaassen C.H. (2012). Genotypic diversity of clinical *Coxiella burnetii* isolates from Portugal based on MST and MLVA typing. Int. J. Med. Microbiol..

[35] Rodolakis A., Héchard C., Caudron C., Souriau A., Bodier C.C., Blanchard B., Camuset P., Devillechaise P., Natorp J.C., Vadet J.P. (2007). Comparison of *Coxiella burnetii* Shedding in milk of dairy bovine, caprine, and ovine herds. J. Dairy Sci..

[36] Tilburg J.J. (2013). Molecular Investigation of the Q Fever Epidemic in the Netherlands. The Largest Outbreak Caused by *Coxiella Burnetii* ever Reported. Ph.D. Thesis.

[37] Mioni M.S.R., Sidi-Boumedine K., Dalanezi F., Joaquim S.F., Denadai R., Teixeira W.S.R., Labruna M.B., Megid J. (2019). New genotypes of *Coxiella burnetii* circulating in brazil and argentina. Pathogens.

[38] Glazunova O., Roux V., Freylikman O., Sekeyova Z., Fournous G., Tyczka J., Tokarevich N., Kovacava E., Marrie T.J., Raoult D. (2005). Coxiella burnetii genotyping. Emerg. Infect. Dis..

[39] Piñero A., Barandika J.F., García-Pérez A.L., Hurtado A. (2015). Genetic diversity and variation over time of *Coxiella burnetii* genotypes in dairy cattle and the farm environment. Infect. Genet. Evol..

[40] Horrevorts A.M., Klaassen H.W.C., Nabuurs-Franssen M.H., Roest H.J.I.J., Tilburg J.H.C. (2011). Genotyping reveals the presence of one predominant C. *burnetii* genotype in milk throughout Europe. Proceedings of the 6th International Meeting on Rickettsiae and Rickettsial Diseases.

[41] Frangoulidis D., Walter M.C., Antwerpen M., Zimmermann P., Janowetz B., Alex M., Böttcher J., Henning K., Hilbert A., Ganter M. (2014). Molecular analysis of *Coxiella burnetii* in Germany reveals evolution of unique clonal clusters. Int. J. Med. Microbiol..

[42] Sulyok K.M., Kreizinger Z., Hornstra H.M., Pearson T., Szigeti A., Dán Á., Balla E., Keim P.S., Gyuranecz M. (2014). Genotyping of *Coxiella burnetii* from domestic ruminants and human in Hungary: Indication of various genotypes. BMC Vet. Res..

[43] Szymańska-Czerwińska M., Jodełko A., Zaręba-Marchewka K., Niemczuk K. (2019). Shedding and genetic diversity of *Coxiella burnetii* in Polish dairy cattle. PLoS ONE.

[44] Hemsley C.M., O’Neill P.A., Essex-Lopresti A., Norville I.H., Atkins T.P., Titball R.W. (2019). Extensive genome analysis of *Coxiella burnetii* reveals limited evolution within genomic groups. BMC Genom..

[45] Lopez-Gatius F., Almeria S., Garcia-Ispierto I. (2012). Serological screening for *Coxiella burnetii* infection and related reproductive per-formance in high producing dairy cows. Res. Vet. Sci..

[46] Agerholm J.S. (2013). *Coxiella burnetii* associated reproductive disorders in domestic animals-a critical review. Acta Vet. Scand..

[47] Koehler L.M., Kloppert B., Hamann H.-P., El-Sayed A., Zschöck M. (2019). Comprehensive literature review of the sources of infection and transmission routes of *Coxiella burnetii*, with particular regard to the criteria of “evidence-based medicine”. Comp. Immunol. Microbiol. Infect. Dis..

[48] Taurel A.-F., Guatteo R., Joly A., Seegers H., Beaudeau F. (2011). Seroprevalence of Q fever in naturally infected dairy cattle herds. Prev. Vet. Med..

